# Nucleolin antagonist triggers autophagic cell death in human glioblastoma primary cells and decreased *in vivo* tumor growth in orthotopic brain tumor model

**DOI:** 10.18632/oncotarget.5990

**Published:** 2015-10-19

**Authors:** Elisabetta Benedetti, Andrea Antonosante, Michele d'Angelo, Loredana Cristiano, Renato Galzio, Damien Destouches, Tiziana Marilena Florio, Anne Chloé Dhez, Carlo Astarita, Benedetta Cinque, Alessia Fidoamore, Floriana Rosati, Maria Grazia Cifone, Rodolfo Ippoliti, Antonio Giordano, José Courty, Annamaria Cimini

**Affiliations:** ^1^ Department of Life, Health and Environmental Sciences, University of L'Aquila, L'Aquila, Italy; ^2^ Department of Cell Biology, Université Paris-Est, UPEC, Créteil, France; ^3^ Laboratoire de Recherche sur la Croissance Cellulaire, la Réparation et la Régénération Tissulaires (CRRET) CNRS, Créteil, France; ^4^ Department of Life Sciences, University of Siena, Siena, Italy; ^5^ Department of Medicine, Surgery and Neuroscience, University of Siena, Siena, Italy; ^6^ Sbarro Institute for Cancer Research and Molecular Medicine and Center for Biotechnology, Temple University, Philadelphia, Pennsylvania, USA; ^7^ National Institute for Nuclear Physics (INFN), Gran Sasso National Laboratory (LNGS), Assergi, Italy

**Keywords:** glioblastoma, autophagy, targeted therapy

## Abstract

Nucleolin (NCL) is highly expressed in several types of cancer and represents an interesting therapeutic target. It is expressed at the plasma membrane of tumor cells, a property which is being used as a marker for several human cancer including glioblastoma. In this study we investigated targeting NCL as a new therapeutic strategy for the treatment of this pathology. To explore this possibility, we studied the effect of an antagonist of NCL, the multivalent pseudopeptide N6L using primary culture of human glioblastoma cells. In this system, N6L inhibits cell growth with different sensitivity depending to NCL localization. Cell cycle analysis indicated that N6L-induced growth reduction was due to a block of the G1/S transition with down-regulation of the expression of cyclin D1 and B2. By monitoring autophagy markers such as p62 and LC3II, we demonstrate that autophagy is enhanced after N6L treatment. In addition, N6L-treatment of mice bearing tumor decreased *in vivo* tumor growth in orthotopic brain tumor model and increase mice survival. The results obtained indicated an anti-proliferative and pro-autophagic effect of N6L and point towards its possible use as adjuvant agent to the standard therapeutic protocols presently utilized for glioblastoma.

## INTRODUCTION

Nucleolin is a ribonucleoprotein over-expressed in highly proliferative cells such as cancer cells [1]. This ribonucleoprotein has been first described as a protein located in the nucleolus, but it is now known that it can shuttle to the nucleoplasm, the cytoplasm and the cell surface of proliferating cells where it plays different functions [1–3]. Accordingly, NCL is expressed at the plasma membrane of tumor cells, a property which is being used as a marker of several human cancer, including glioblastoma [4]. At cell surface, NCL binds to several ligands including growth factors such as midkine, pleitrophin, hepatocyte growth factor and vascular endothelial growth factor [5–7].

Accordingly, various ligands targeting cell-surface nucleolin have been used to block tumor growth and angiogenesis, including endostatin [8] aptamer AS1411 [9], F3 tumor-homing peptide [10] and the multivalent pseudopeptide N6L [11]. N6L is composed of a short template, rich in lysine residues on which six pseudotripeptide Lysψ(CH_2_N)-Pro-Arg is covalently anchored [12].

N6L specifically targets cancer cells, exhibiting anti-tumor activity in various human tumor cell lines derived from mammary, colorectal carcinoma, melanoma and glioblastoma (GB) [12]. This therapeutic peptide is currently in phase I/IIa clinical trials (NCT01711398).

Since mature brain expresses important levels of NCL during the differentiation of neural cells, it is reasonable to suggest that NCL has a fundamental role in mediating signals by extracellular matrix molecules, and furthermore contributes to the differentiation and maintenance of neural tissue [13]. Our previous data showed that in gliomas, surface NCL localization increased with the malignancy grade suggesting that NCL could be considered as a histopathological marker for glioma grading [14], a tumor difficult to be early diagnosed due to the faint appearance of clear symptoms [15]. On the basis of these results, NCL represents a potential tool for targeted therapy for gliomas, and thus in this work the effects of N6L on human GB cells in primary culture prepared form post-surgical specimens and also in *in vivo* assays were investigated.

## RESULTS

### N6L inhibits GB cell growth *in vitro* with different sensitivity depending on NCL localization and N6L internalization

**Figure 1:**
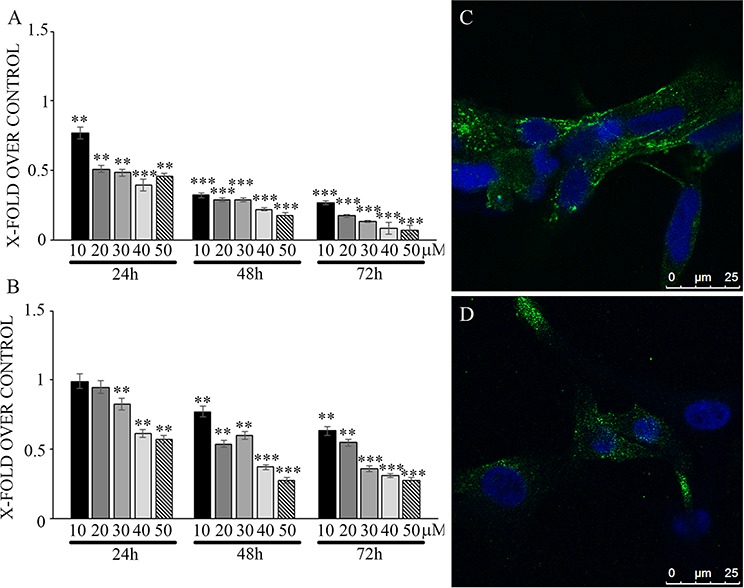
Viability assay on glioblastoma primary cultures, more sensitive (panel A) and less sensitive cells (panel B) upon treatment with different N6L concentrations for different timepoints Data are reported with respect to control untreated cells. The experiment reported is representative of 4 experiments performed in quadruplicate. Data are mean ± SE; **,*p* < 0.005; ***p < 0.0005. In **C** and **D** nucleolin immunolocalization in more sensitive and less sensitive cells, respectively.

**Figure 2:**
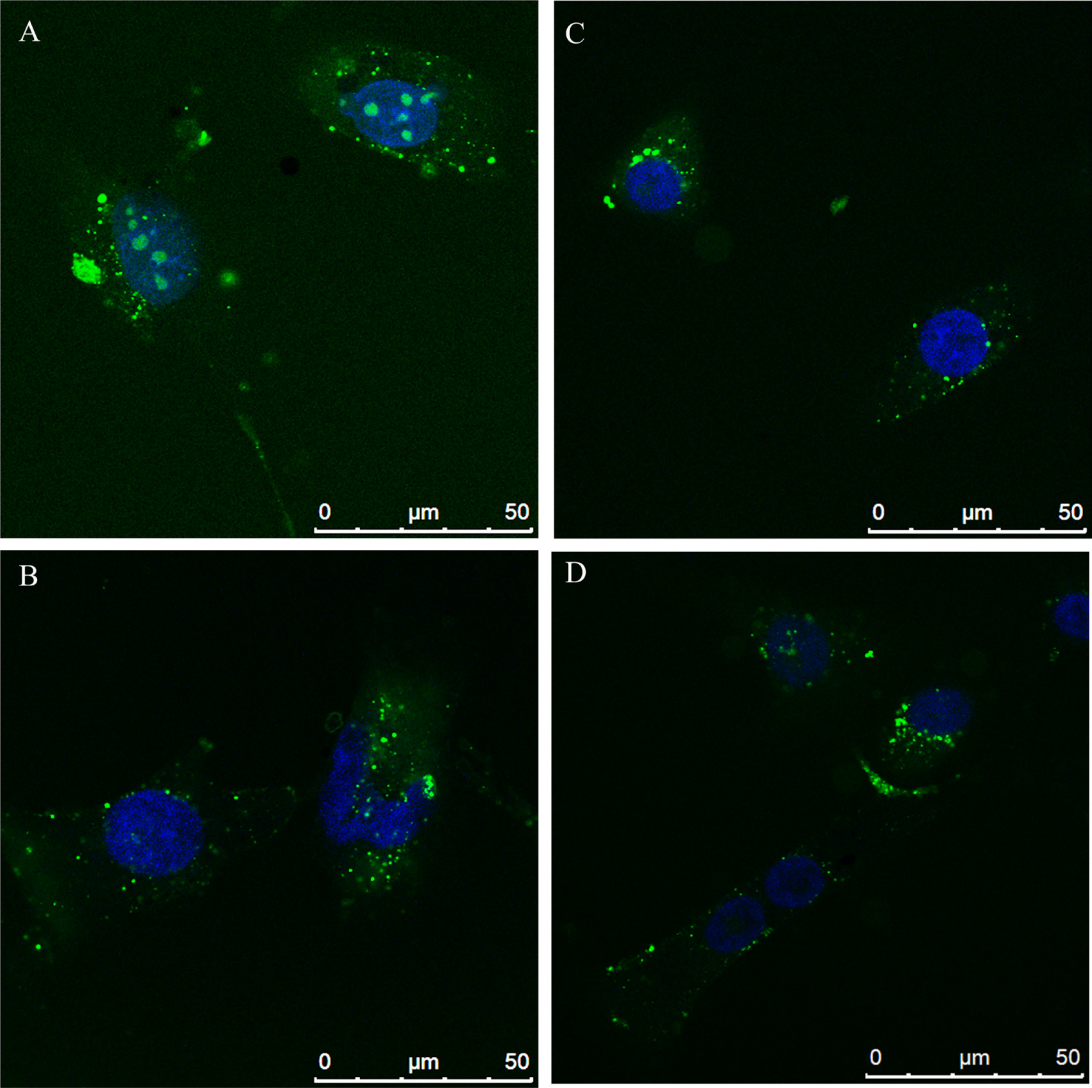
N6L internalization by Alexafluor 488-N6L (fN6L) in the more responsive cultures **A.** and **B.** and in the less responsive ones **C.** and **D.**

Effects of N6L on GB cells were studied using primary cultures derived from surgical specimens obtained from 15 patients. As shown in Figure 1, N6L decreases cell viability in a time- and concentration-dependent manner. However, different sample sensitivity to the treatment was observed according to the patient's source (Figure 1A and 1B). In fact, some samples were highly sensitive to N6L other less sensitive with a GI_50_ ranging from 1.97 μM to 30 μM (Figure 1A). Possible correlation between cells sensitivity to N6L and nucleolin expression rate has been next investigated. Nucleolin is abundantly expressed in the cytoplasm and membrane of the more N6L responsive cultures (Figure 1C), while it is less abundant in cells which are less sensitive to N6L (Figure 1D). In order to study the N6L internalization into the cell cytoplasm, fluorescent N6L (fN6L) was used (Figure 2). When GB cells were challenged with 40 μM fN6L, the more responsive cultures showed the peptide strongly localized in the cytoplasm and nucleolus (Figure 2A), whereas in the less responsive ones fN6L was less abundantly present in the cytoplasm and not localized in the nucleolus (Figure 2C). When cells were challenged with 10 μM fN6L, the nucleolar positivity was lost in both culture types, whereas in the more sensitive cultures the membrane/cytoplasmatic positivity was more apparent than in less sensitive cultures (Figure 2B and 2D, respectively). These data indicate a more effective internalization in the nucleolus and cytoplasm of N6L in the more responsive cells, suggesting that the effect of N6L occurred via its internalization.

Due to the differences of sensitivity and according to the different GI_50_, the subsequent experiments were performed using N6L at 10 μM in the responsive cultures and at 40 μM in the less responsive ones. However, since behaviors of the different parameters studied upon N6L challenge (evaluated vs the respective control) were the same in the different patient populations, the results obtained in the different cultures (more responsive and less responsive) were pooled and statistically analyzed.

### N6L inhibits cell cycle of GB cells *in vitro*

**Figure 3:**
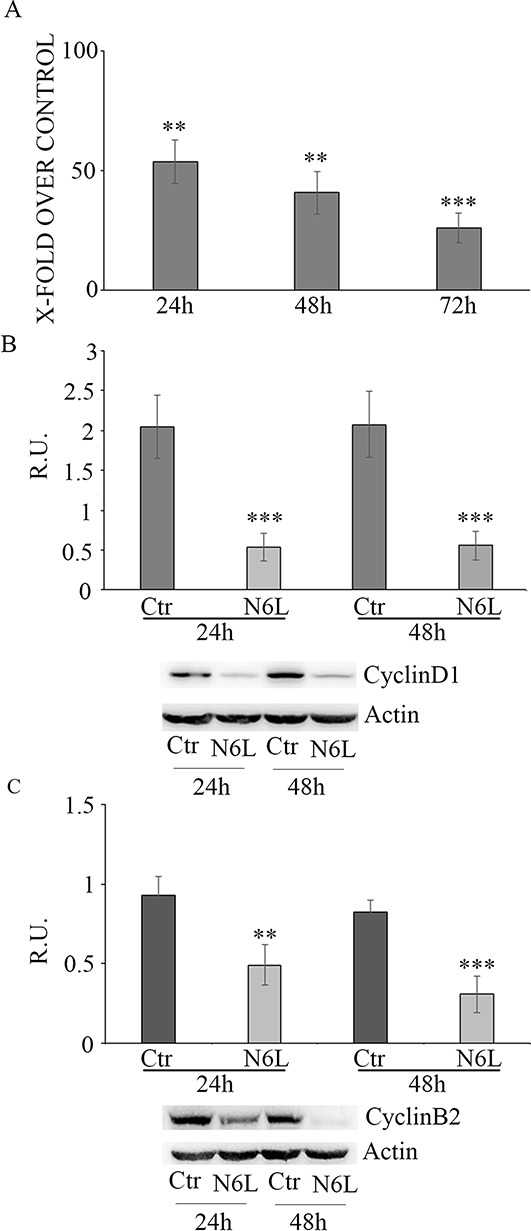
**Panel A:** Proliferation assay in control and N6L-treated cells, evaluated by BrdU incorporation at different timepointsThe data are expressed as percentage of the relative control. The experiment reported is representative of 4 experiments for each culture performed in quadruplicate. Data are mean ± SE. **, *p* < 0.005; ***p < 0.0005. In **panel B:** western blotting analysis for cyclin D1 in control and N6L-treated cultures for 24 h and 48 h. A representative blotting is shown; the densitometric analysis is the mean ± SE of 4 different experiments for each culture. ***, *p* < 0.0005; **Panel C:** western blotting analysis for cyclin B2 in control and N6L-treated cultures for 24 h and 48 h. A representative blotting is shown, the densitometric analysis is the mean ± SE of 4 different experiments for each culture. **, *p* < 0.005; ***, *p* < 0.0005.

**Figure 4:**
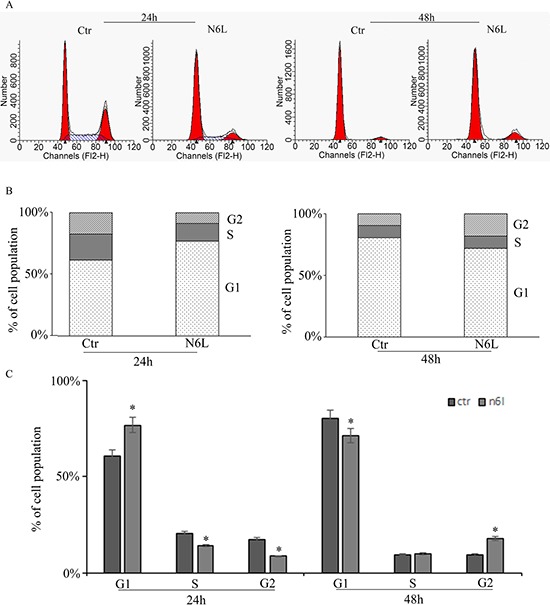
Cell cycle analysis measured by cytofluorimetry on glioblastoma primary cultures upon treatment with N6L for 24 and 48 h The experiment reported is representative of quadruplicate experiments. The **panel A:** shows the original cytofluorimetric profiles, the **panels B:** and **C:** show the analysis of the cell population distribution along the cell cycle phases in control and treated conditions. Data are mean ± SE. *, *p* < 0.05.

Effect of N6L on GB cell proliferation was evaluated by BrdU incorporation (Figure 3). Upon N6L challenge, BrdU incorporation was strongly decreased in time dependent manner (Figure 3A). Accordingly, N6L dramatically decreased both cyclin D1 (Figure 3B) and cyclin B2 (Figure 3C) expression. To complete this data, cytofluorimetric analysis for cell cycle was carried out (Figure 4A and 4B). Cell cycle analysis using PI staining and FACS analysis showed that after 24 h, N6L was able to induce an increase of cells in G1 phase with concomitant decrease of cells in the S and G2 phases, which is in agreement with the decrease in the expression of cyclin D1. Upon 48 h of treatment, control cells reached confluence and were almost blocked in G1, while the treatment determined a block in G2/M phase, in agreement with the decrease in the expression of cyclin B2 observed (Figure 4C). These results showing that N6L is able to perturb the cell cycle prompted us to analyze the mechanism of action of N6L.

### N6L inhibited cell migration of GB cells *in vitro*

**Figure 5:**
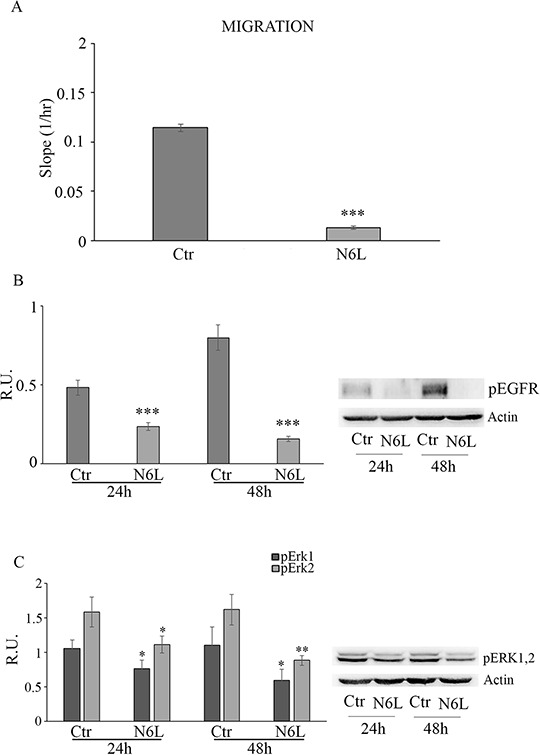
**Panel A:** cell migration in control and N6L-treated cellsThe experiment reported is representative of 4 experiments for each culture performed in quadruplicate. Data are mean ± SE. ***, *p* < 0.0005. **Panel B:** western blotting analysis for p-EGFR in control and N6L-treated cultures for 24 h and 48 h. A representative blotting is shown; the densitometric analysis is the mean ± SE of 4 different experiments for each culture. ***, *p* < 0.0005; **Panel C:** western blotting analysis for *p*-ERK1,2 in control and N6L-treated cultures for 24 h and 48 h. A representative blotting is shown; the densitometric analysis is the mean ± SE of 4 different experiments for each culture. *, *p* < 0.05; **, *p* < 0.005.

Since N6L has been shown to inhibit metastasis [12], we next investigated the effect of N6L on the migration of GB cells. In figure 5A cell migration in control and treated cultures is reported; upon N6L challenge cell migration was strongly decreased already at 24 h in all culture analyzed. Consistently, N6L dramatically decreases both the active form of ErbB1 receptor (EGFR) (Figure 5B) and ERK1/2 (Figure 5C), known to be implicated in proliferation, migration and invasion of glioma cells [16–17].

### N6L induced autophagy in glioblastoma cells *in vitro*

**Figure 6:**
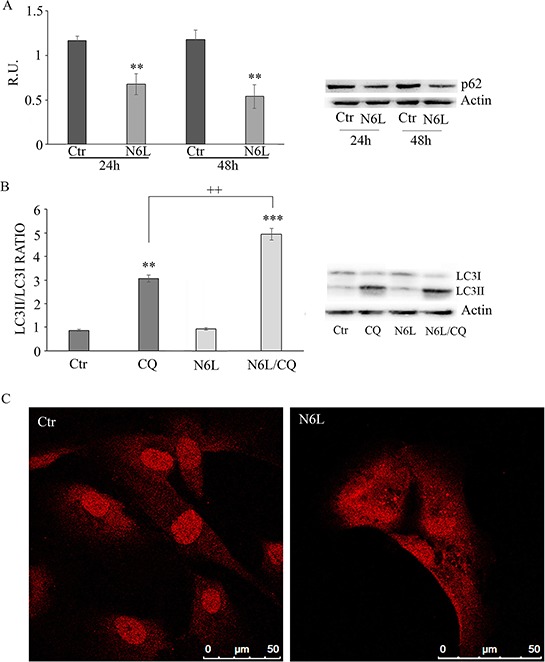
**A:** western blotting analysis for *p*-62 in control and N6L-treated cultures for 24 h and 48 h. A representative blotting is shown; the densitometric analysis is the mean ± SE of 4 different experiments for each culture. **, *p* < 0.005. **B:** autophagic flux evaluation by CQ treatment in the presence/absence of N6L by western blotting analysis for LC3II and LC3I. A representative blotting is shown; the densitometric analysis, representing the ratio LC3II/LC3I is the mean ± SE of 4 different experiments for each culture. **p < 0.005; ***, *p* < 0.0005; ++, *p* < 0.005, N6L/CQ versus CQ **C:** LC3 immunolocalization in control and N6L-treated cells.

Since the nucleosome concentration was not significantly modulated upon N6L treatment (data not shown) suggesting that the effect of N6L occurs through a mechanism different from apoptosis, the autophagic markers were investigated including p62, LC3I and II (Figure 6). Since it is known that p62 is selectively incorporated into autophagosomes through direct binding to LC3 and that it is efficiently degraded by autophagy [18], generally the total cellular levels of p62 inversely correlate with the autophagic activity. Accordingly, in our experimental conditions, As reported in figure 6A, p62 was significantly decreased by N6L at 48 h, thus indicating that N6L is able to induce autophagic death. Accordingly, the ratio LC3 II/LC3I was increased by treatment with chloroquine (CQ)+ N6L (Figure 6B). These findings were further confirmed by the strong increase, upon treatment, of the “punctata” form of LC3, a marker of autophagosomes number (Figure 6C). It is worth noting that LC3 is present also in the nucleus in untreated cells (Figure 6C) and that it is known that deacetylation of nuclear LC3 drives autophagy initiation, inducing cytoplasmic translocation of LC3 [19]; accordingly, upon N6L treatment, LC3 changed its localization, moving from the nucleus to the cytoplasm (Figure 6C).

**Figure 7:**
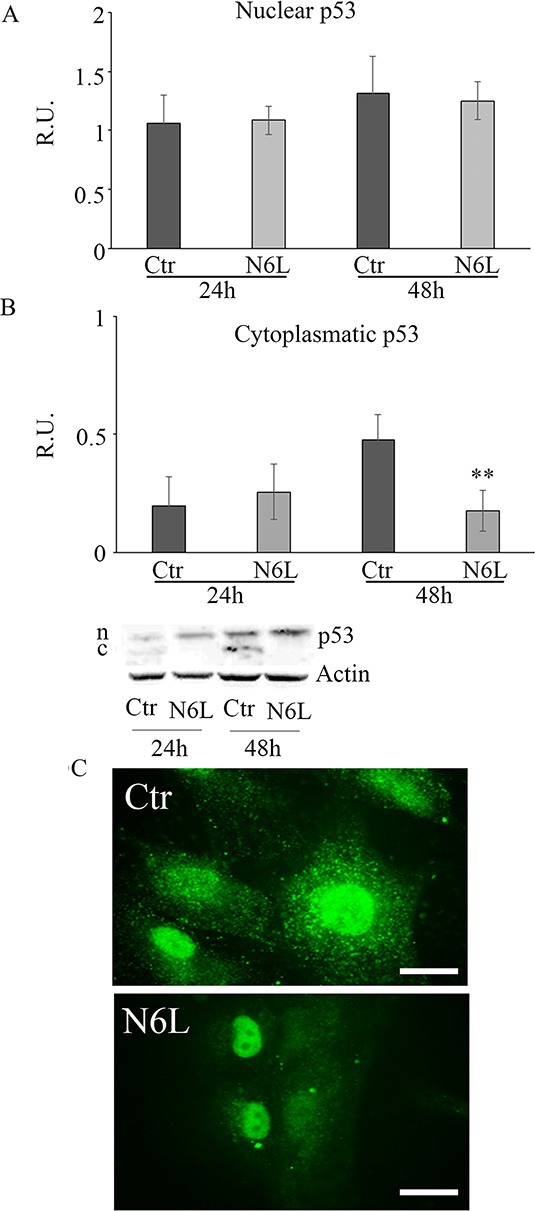
**A:** western blotting analysis for nuclear *p*-53 in control and N6L-treated cultures at 24 h and 48 h. A representative blotting is shown; the densitometric analysis is the mean ± SE of 4 different experiments for each culture. **B:** western blotting analysis for cytosolic *p*-53 in control and N6L-treated cultures for 24 h and 48 h. A representative blotting is shown; the densitometric analysis is the mean ± SE of 4 different experiments for each culture. **, *p* < 0.005. **C:** *p*-53 immunolocalization in control and N6L-treated cells.

Since it is known that cytosolic p53 form inhibits authophagic cell death, the cytosolic and nuclear forms of p53 have been studied (Figure 7). Upon treatment, the p53 nuclear content is not affected (Figure 7A), while the cytoplasmic form is significantly decreased at 48 h (Figure 7B). This is also apparent in immunofluorescent studies (Figure 7C), where the immunofluorescent localization of p53 upon treatment showed an evident nuclear localization paralleled by the decrease of cytoplasmic p53.

### *In vivo* effects of N6L

**Figure 8:**
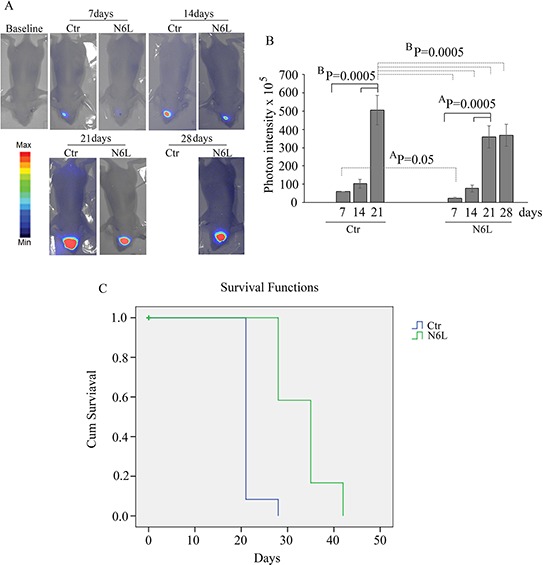
*in vivo* studies performed on BALB/c-nu/nu athymic mice injected intracranially with U87 LUC cells and treated with 10 mg/kg bw of N6L for 5 days/week from day 1 post-cell inoculation for 6 weeks In Panel **A:** representative images of the bioluminescence in control and N6L-treated mice is reported; In Panel **B:** quantification of the bioluminescence signal recorded at the1st, 2nd, 3rd, and 4th week from cell injection. The BP values shown are the results of the ANOVA for each group, while AP values shown are the results of the ANOVA for control group versus treated group. In Panel **C:** the survival function obtained by Kaplan-Meier survival plot of N6L treated and sham-treated control animals. The mean survival times were 33.3+/–1.5 and 21.6+/–0.6 days, respectively, for the treated and control groups (mean+/–SE, *n* = 12). There was a statistically significant increase life span in the treated animals (*P* = 0.0001 Mantel-Cox log rank test).

Finally, *in vivo* studies were performed on BALB/c-nu/nu athymic mice injected intracranially with U87 LUC cells. The bioluminescence data, analyzed weekly, showed a significant decrease of tumor growth in N6L treated mice (Figure 8A and 8B). The mean light intensity detected on day 21 for the group of mice treated with N6L was lower than the control group (*p* = 0.0005). In addition, the survival function shows that N6L was able to increase mice survival until 6 weeks while 90% of untreated mice were dead at 3 weeks (Figure 8C).

## DISCUSSION

Many studies showed that cell-surface NCL plays a crucial role in tumor growth as well as in angiogenesis [10–11; 20]. Recently, our group has shown that surface NCL is increased with the malignancy grade of gliomas, suggesting that it may constitute a histopathological marker for glioma grading and a potential tool for targeted therapy [14]. GB is the most invasive and aggressive brain tumor in humans, and despite the latest chemical and radiation approaches, it is still poorly sensitive to these treatments and is considered an incurable disease [15]. Therefore, functional blockade of cell surface NCL with an antagonist such as N6L may represent an innovative therapy against this kind of tumor.

In this study, we investigated the biological role of N6L on primary cultures of human GB, that represent much more than the cell lines, the individual response to pharmacological treatment, due to their different biomolecular profile. In agreement with previously published data [12], we found that N6L significantly reduced cell viability of human GB primary culture at any time and concentration considered. It is noteworthy that the sensitivity of the N6L depends on the NCL expression profile of each patient. In fact, many cultures appeared extremely sensitive to the treatment with N6L, while other cultures from different patients appeared less responsive to N6L. This different response is linked to the different expression of surface/cytoplamic NCL in the two sets of GB cultures, where NCL was abundantly present in the responsive cultures, while it was less abundant in the other cultures. This data was further confirmed by the N6L internalization study, showing that N6L was much more internalized in the responsive cultures than in the less responsive ones. The internalization of N6L was paralleled by the significant inhibition of cell proliferation accompanied by a dramatically decrease of cyclin D1 and cyclin B2. In fact, a blockage in G1 phase at 24 h and in G2/M at 48 h were promoted by N6L treatment.

In our experimental conditions, unlike previous experiments on different cancer cells lines [12], we did not observed apoptosis induction upon N6L treatment, nor by cytofluorimetry or nucleosome concentration (data not shown). This may be due to the different tumor considered in the previous study but also to the fact that we use primary cultures from GB patients. Moreover, we showed that N6L induced autophagic cell death as demonstrated by the increased protein levels of LC3II in the presence of CQ and by the decreased protein levels of p62, the two key components in the formation of autophagosomes, and by the strong increase of the “punctata” form of LC3 and by the LC3 redistribution from nucleus to cytoplasm.

Interestingly, N6L induced autophagic process through a decrease of the cytoplasmic p53. In fact, it is known that p53 plays a dual role in the control of autophagy. On one hand, nuclear p53 induces autophagy through its transcriptional effects, on the other hand, cytoplasmic p53 acts as a master repressor of autophagy [21]. Autophagy is a conserved mechanism that cells utilize to degrade intracellular long-lived proteins and organelles through lysosome-mediated degradation. Depending on the stimulus and the tumor cell type, autophagy can either act to protect tumor cells from a toxic stress or can facilitate the toxicity of the stress [22]. In agreement with the role of autophagy in the enhancement of the toxicity level of many anticancer compounds in tumor cells, N6L increased LC3 II processing and decreased p62. Interestingly, it has been reported that p62 itself has oncogenic functions [23–25]. N6L, therefore, by triggering autophagy, may inhibit tumorigenesis by limiting p62 availability.

Since it was demonstrated that NCL interacts with EGFR leading to receptor dimerization and activation [26] with consequent promotion of proliferation and migration, we decided also to investigate the effects of N6L on EGFR activation. A dramatically decrease of the activation of EGFR and pERK1/2 which is the downstream effector, was observed. Similar data has been observed with a polyplexed form on N6L tested on U87-MG line cells (unpublished data). On the basis of the implication of EGFR not only in proliferation, but also in migration of glioma cells [16–17], we also assayed cell migration and found that it was strongly decreased upon N6L treatment. Our results are in agreement with those of Farin and coll., that suggested the use of aptamer-targeting NCL on GB cell lines, as a new strategy to inhibit the activation of EGFR- and Ras-driven cancers [27].

We therefore propose N6L as possible adjuvant agent to the standard therapeutic protocols presently utilized for GB. In fact, in preliminary *in vitro* experiments (Supplementary Figure S1), we observed that N6L, in combination with Temozolomide (TMZ), resulted effective in significantly reducing cell viability with respect to N6L and TMZ alone. Moreover we also observed that N6L was more effective in inducing cell death than TMZ, even if it was administered at 10–40 μM with respect to the higher TMZ concentration utilized (1 mM). Finally, *in vitro* data were confirmed by *in vivo* studies, showing that N6L decreases tumor growth and increases mice survival until 6 weeks.

All together our results, obtained in different sets of primary culture of GB derived from surgical specimens of patients, show for the first time a strong pro-autophagic, anti-proliferative and anti-migrative effect of N6L. Interestingly, recent data showed that treatment of glioma cells with autophagy inducing agents such as Tamoxifen increases the efficacy of oncolytic adenoviruses treatment [28]. A different study demonstrated that combined treatment including those targeting nucleolin and Ras may represent an additional opportunity for inhibiting GB [29]. According to these data, it is tempting to speculate that combined treatments of N6L with molecules targeting overexpressed oncogenes represent an interesting way to treat cancer patient.

## MATERIALS AND METHODS

### Materials

All materials were of the highest analytical grade and were from Sigma Chemical CO (St. Louis, CO, USA) unless otherwise stated. D-luciferin monosodium salt both were purchased from Thermo Scientific (Pierce Biotechnology, Rockford, IL, USA). N6L and N6L-alexafluor 546 (fN6L) where synthetized according to a procedure previously described [14]. Molecular weights standard and blocking solution were purchased from Bio-Rad Laboratoires (Hercules, USA). SuperSignal West Pico Chemiluminescent Substrate was purchased from Thermo Scientific (Pierce Biotechnology, Rockford, IL, USA).

### Patient population

This study was ethically approved (Hospital Ethics Committee), and all patients were voluntary signing an informed consent. Newly diagnosed GB patients (43 years to 73 years old, mean age of 60 years) were surgical resected at the Department of Neurosurgery, San Salvatore Hospital, L'Aquila, Italy. Patients followed complete clinical and neurological evaluation at the admission in order to evaluate clinical conditions and Karnofsky Performance Status, including neuro-radiological investigation by C.T. scan without contrast enhancement, MRI with and without gadolinium, technetium 99 MIBI brain APECT. Tumor biopsies used in this study were from patients whose lesions were suitable for gross total removal. Surgical removal starts from the edematous brain surrounding the tumor and moves from the borders avoiding initial lesion debulking. Surgery was performed by Image Guided Surgery.

### Cell culture

Individual tumor biopsies (15 patients) excluding necrotic fragments were maintained in culture medium and addressed in ice to our laboratory. The fragments were then rinsed with Hank's balanced salt solution (HBSS), the necrotic areas and red endothelial parts were moved aside. Primary cell culture was established as previously described [12]. U87-LUC cells, were kindly provided by Dr. David Gillespie (Huntsman Cancer Institute Salt Lake City, UTAH 84010) and were cultured in DMEM supplemented with 10% FBS containing 50 UI/ml pennycillin-streptomycin.

### Cell treatments

Cells were seed at a density of 5000 cells/cm^2^ and all treatments were performed one day after plating. For cell viability, N6L was tested at 24, 48 and 72 hours at increasing concentrations, ranging from 10 μM to 50 μM in DMEM containing 5% FBS. Temozolomide (TMZ), was dissolved in DMSO and the cell viability was tested at 72 h. Control cells were treated with DMSO (vehicle) in DMEM containing 5% of FBS.

### N6L internalization

Cells were seed at a density of 5000 cells/cm^2^ and one day after plating were treated with 40 μM or 10 μM of N6L-Alexa 488 (fN6L) in DMEM containing 5% FBS, for 30 minutes at 37°C in 5% CO2, 95% air atmosphere. Subsequently, the cells were extensive washed with PBS and fixed for 10 min at RT in 4% paraformaldehyde in PBS. Cells were mounted with Vectashield mounting medium containing DAPI and photographed at confocal microscope LEICA TCS SP2 (LEICA, Mannheim, Germany).

### Cell viability assay

Cells were seeded (2500 cells/well) in a 96 wells plate in presence or absence of treatment (every treatment was performed in triplicate) and incubated for indicated time in the figure. At expiration of incubation period cell viability was determined using Cell Titer One Solution Cell Proliferation Assay (Promega, Lyon, France) reading the absorbance at 492 nm, in a spectrophotometric microplate reader Infinite F200 (Tecan, Männedorf, Swiss). The results are expressed as ratio between the absorbance of treated cells compared to absorbance of control untreated cells. GI_50_ was calculated fitting the experimental data using the software Grafit 5.0 (Erithacus Software, UK).

### Cell proliferation assay

Cells were seeded in a 96 wells plate (2500 cells/well). Cell proliferation was determined using 5′-Bromo-2′-deoxyuridine (BrdU) labeling and detection kit (Roche, Penzberg, Germany) reading the absorbance at 405 nm, measured in a spectrophotometric microplate reader (Infinite F200 Tecan). The results are expressed as ratio between the absorbance of treated cells compared with absorbance of control untreated cells.

### Autophagy flux

Cells were seeded at a density of 5000 cells/cm^2^. One day after plating, cells were treated with 20 μM chloroquine diphosphate salt (CQ), N6L (10 μM or 40 μM) or both N6L and CQ for 6 hours in DMEM containing 5% FBS. Control cells were treated D-mannitol (vehicle) in DMEM containing 5% FBS. The protein levels of LC3 were assayed by Western blotting and immufluorescence experiments as described below.

### Protein assay

Protein were assayed by the Pierce BCA Protein Assay kit (Rockford, IL, USA) reading absorbance at 562 nm (Infinite F200 Tecan).

### Western blotting

For Western blotting, cell lysates in ice-cold RIPA buffer were centrifuged and the supernatants were assayed for protein content. About 20 to 30 μg of proteins were fractionated on 7.5–15% polyacrylamide gel and transferred onto PVDF membrane from Millipore Corporation (Billerica, MA, USA). Nonspecific binding sites were blocked for 1 hour at RT in 20 mM Tris-HCl (pH 7.4) buffer, 55 mM NaCl and 0.1% Tween 20 containing 5% non-fat dry milk (blocking buffer). Membranes were then incubated overnight at 4°C with primary antibody diluted in blocking buffer. Primary antibodies used are anti-LC3, anti-cyclin D1, anti-p62, anti-actin and anti-pERK1,2 antibodies from Sigma Aldrich (St. Louis, USA), anti-p53 and anti-pEGFR antibodies from Santacruz Biotectnology (SantaCruz, CA, USA), anti-cyclin B2 was from Abcam (Cambridge Science Park, Cambridge, UK). All these antibodies are dissolved in blocking solution. After extensive washings and incubation with the respective horseradish peroxidase-labeled secondary antibodies, protein presence was visualized by enhanced chemiluminescence reaction from Pierce Biotechnology (Rockford, IL, USA). Band relative densities obtained using Alliance 4.7 UVITEC (Cambridge, UK) were normalized to actin and values were given as relative units (R.U.).

### Immunofluorescence

Cells plated on poly-L-lysine coated coverslip, were washed twice with PBS, fixed for 10 min at RT in 4% paraformaldehyde in PBS and permeabilized in PBS containing 0.1% Triton X-100 for 10 min at RT. Nonspecific binding sites were blocked for 30 min with 3% BSA in PBS (incubation buffer). According to the experiment, cells were then incubated with either rabbit anti-LC3, anti-nucleolin or anti-p53 in incubation buffer overnight at 4°C. For nucleolin staining the permeabilizzation step was omitted. After extensive washings with PBS the cells were incubated with AlexaFluor 488 or 546 secondary antibodies 30 min at RT. Cells were then washed and mounted with Vectashield mounting medium from Vector Laboratories (Burlingame, CA, USA) containing DAPI. Confocal image were then obtained using microscope LEICA TCS SP2 (LEICA, Mannheim, Germany). Regarding p53 staining, cells were photographed at florescence microscope AXIOPHOT (Zeiss microscope, Jena, Germany).

### Cell migration assay

Cell migration assay has been carrier with RTCA DP Instrument (ACEA Biosciences, San Diego, CA, USA) using 16-well plates which has an upper chamber, sealed at the bottom with a microporous (8 μm of diameter) PET membrane coated with 0.1% gelatin using FBS as chemoattractant.

### Animals

Procedures involving animal care were conducted in conformity with national and international laws and policies (EEC Council Directive 86/609, OJ L 358, 1, Dec. 12,1987; Italian Legislative Decree (Gu n. 61,14/03/2014); NIH guide for the Care and Use of Laboratory Animals, NIH Publication No. 85-23, 1985), and were approved by the Institutional Review Board of the University of L'Aquila

### Orthotopic implantation of tumor cells and treatment

U87-LUC cells were injected (1.5 × 10^5^ in 3 μL HBSS) into the right striatum (coordinates : 1 mm lateral to the middle line and depth 3.5 mm) through a burr hole in the skull using a 10-μl Hamilton syringe to deliver tumor cells to a intraparenchymal depth of 4-week-old female BALB/c-nu/nu athymic mice (Charles River Laboratories France) anesthetized with a ketamine/xylazine cocktail solution. The mice were treated by intraperitoneal injection with 10 mg/kg body weight of N6L or vehicle as control for 5 days/week from day 1 post-cell inoculation for 4 weeks. Tumor growth was monitored and measured via bioluminescence imaging *in vivo* by intraperitoneal injection of 100 mg/kg of body weight of D-luciferin using the Aequoria 2D luminescence imaging system according to the recommended procedure (Hamamatsu Photonics, Naka-ku, Japan). Animals were daily monitored for cachexia (evaluated by body weight waste), behavior and survival. Animals that lost about 20% of the body weight were euthanized.

### Kaplan-Meier survival plot

Protocol assay endpoints were the time of death or euthanasia when the animals lost 20% of body weight. *P*-values and survival analyses were calculated based on Log-rank statistical method and presented in Kaplan-Meier plots generated using SPSS software.

### Statistical analysis

For statistical analysis samples were processed by SPSS software. Statistical analysis of two population means was performed by the unpaired Student's *t* test, while statistical differences comparing multiple means were analyzed by the analysis of variance (ANOVA) followed by Scheffe's post hoc test” analysis. *P < 0.05; **P < 0.005, **P < 0.0005. Data were expressed as mean ± SE of 4 separate experiments.

## SUPPLEMENTARY FIGURE


