# Supplementary material for: Nucleolin antagonist triggers autophagic cell death in human glioblastoma primary cells and decreased in vivo tumor growth in orthotopic brain tumor model
Source: Oncotarget. 2015 Oct 19;6(39):42091–104. doi: 10.18632/oncotarget.5990 (PMC4747212; doi:10.18632/oncotarget.5990)
Supplement: Supplementary file 1 [file oncotarget-06-42091-s001.pdf]

SUPPLEMENTARY FIGURE

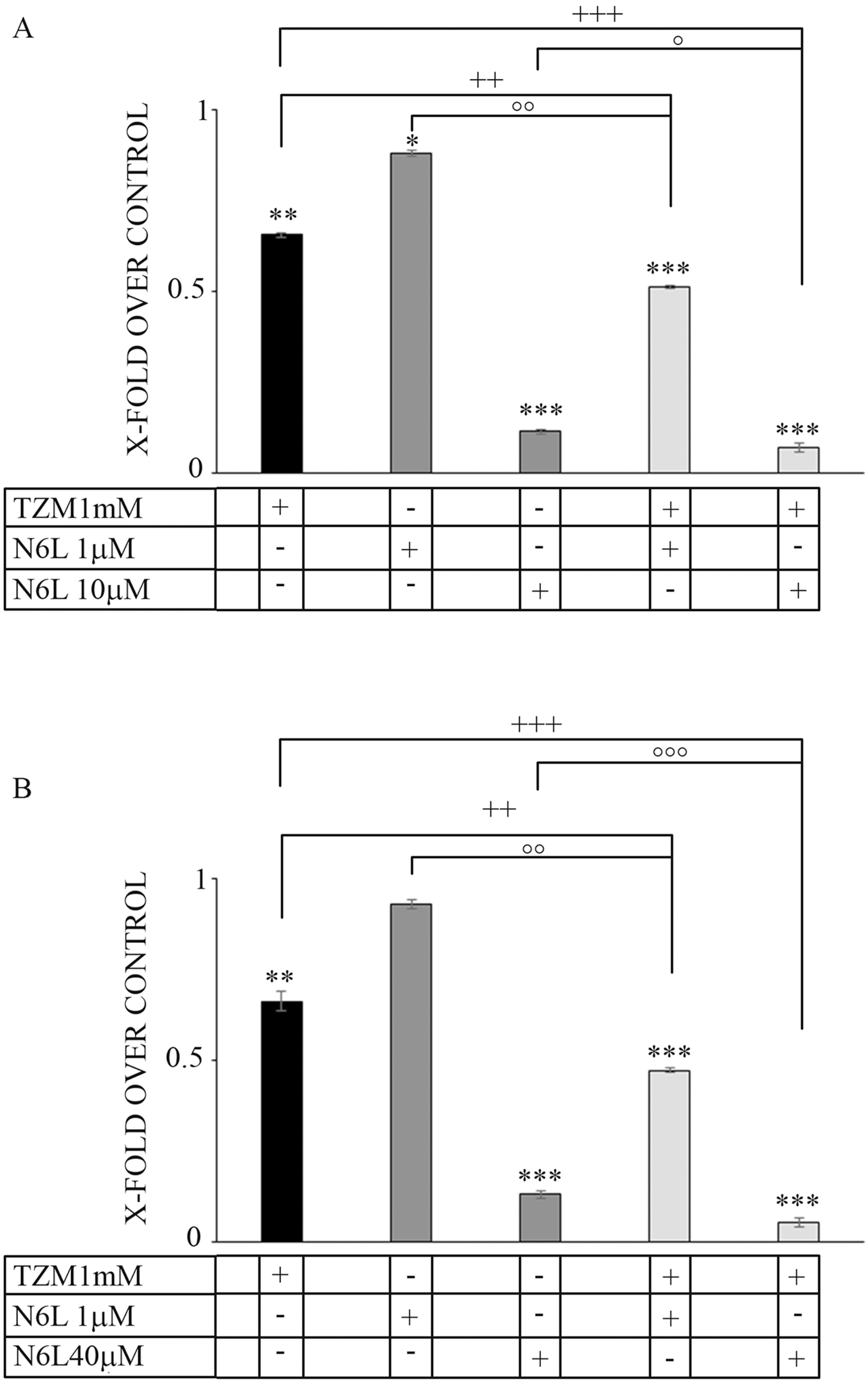

Supplementary Figure S1: Cell viability assay in more sensitive Panel A. and less sensitive cultures Panel B. exposed to N6L, in the presence of temozolomide (TMZ). Data are expressed with respect to the relative control. Data are mean ± SE of 4 different experiments run in quadruplicate. *, $p < 0.05$; **$p < 0.005$; ***, $p < 0.0005$; +, $p < 0.05$; ++, $p < 0.005$; +++, $p < 0.0005$, N6L/TMZ versus TMZ alone. °, $p < 0.05$; °°, $p < 0.005$; °°°, $p < 0.0005$, N6L/TMZ versus N6L alone.
